# Supplementary material for: The Systems Biology Research Tool: evolvable open-source software
Source: BMC Syst Biol. 2008 Jun 29;2:55. doi: 10.1186/1752-0509-2-55 (PMC2446383; doi:10.1186/1752-0509-2-55)
Supplement: Additional file 1 — SBRT Archive. An archive of the current version of the Systems Biology Research Tool. [file 1752-0509-2-55-S1.zip › sbrt-1.4.0/doc/developers_guide/api/sbrt/kernel/fba/proc/CatalystDeletionProcess.html]

CatalystDeletionProcess


|  |  |  |
| --- | --- | --- |
| **Overview** \| **Package** \| **Class** \| **Use** \| **Tree** \| **Deprecated** \| **Index** \| **Help** | |  |
| PREV CLASS   **NEXT CLASS** | **FRAMES**    **NO FRAMES**     **All Classes** |  |
| SUMMARY: NESTED \| FIELD \| CONSTR \| METHOD | DETAIL: FIELD \| CONSTR \| METHOD |  |


---


## sbrt.kernel.fba.proc Class CatalystDeletionProcess

```
java.lang.Object
  sbrt.kernel.fba.proc.FbaProcess<FbaOptComputer.Results>
      sbrt.kernel.fba.proc.CatalystDeletionProcess
```

**All Implemented Interfaces:**: KernelProcess<FbaOptComputer.Results>, MonitoredProcess<FbaOptComputer.Results>

---

``` public final class CatalystDeletionProcess extends FbaProcess<FbaOptComputer.Results> implements MonitoredProcess<FbaOptComputer.Results> ```

This class is used to perform catalyst deletion experiments.

**Author:**
:   This class was written and documented by Jeremiah Wright
    while in the Wagner lab.

---

**Constructor Summary**

`CatalystDeletionProcess(RxnDeletionComputer computer)`
  
          Constructs a new catalyst deletion experiment.


| **Method Summary** | |
| --- | --- |
| `IrrevRxnFluxome` | `getFluxome()`             Returns the fluxome used by the reaction deletion computer. |
| `MathExpr<java.lang.String>` | `getObjectiveFunction()`             Returns the current objective function. |
| `OptimizationSense` | `getOptimizationSense()`             Returns the current optimization sense. |
| `ProgressMonitor` | `getProgress()`             Returns a progress monitor for this experiment. |
| `static java.util.Set<java.lang.String>` | `getRxnNames(java.util.Set<java.lang.String> catalysts, IrrevRxnFluxome fluxome)`             Returns the set of names of reactions whose activity depends on the provided set of catalysts. |
| `double` | `getZeroCutoff()`             Returns the current zero cutoff used by the reaction deletion computer. |
| `void` | `run(ResultsManager<FbaOptComputer.Results> resultsManager)`             Runs this process and adds the generated results to the provided results manager via its `ResultsManager.addData(Object)` method. |
| `void` | `setCatalystDelSource(java.lang.Iterable<java.util.Set<java.lang.String>> deletionSource)`             Sets the catalyst deletion input source. |
| `void` | `setObjectiveFunction(MathExpr<java.lang.String> objective)`             Sets the objective function to the provided mathematical expression. |
| `void` | `setOptimizationSense(OptimizationSense sense)`             Sets the optimization sense to the provided value. |
| `void` | `setZeroCutoff(double zeroCutoff)`             Sets the zero cutoff to the provided value. |

**Methods inherited from class sbrt.kernel.fba.proc.FbaProcess**

`toString`

**Methods inherited from class java.lang.Object**

`clone, equals, finalize, getClass, hashCode, notify, notifyAll, wait, wait, wait`

**Constructor Detail**

### CatalystDeletionProcess

```
public CatalystDeletionProcess(RxnDeletionComputer computer)
```

:   Constructs a new catalyst deletion experiment.

    **Parameters:**: `computer` - the computer with which all results will be computed.


**Method Detail**

### getFluxome

```
public IrrevRxnFluxome getFluxome()
```

:   Returns the fluxome used by the reaction deletion computer.

    :   **Specified by:**: `getFluxome` in class `FbaProcess<FbaOptComputer.Results>`
    :   **Returns:**: the fluxome used by the reaction deletion computer.

---


### getZeroCutoff

```
public double getZeroCutoff()
```

:   Returns the current zero cutoff used by the reaction deletion computer.

    :   **Returns:**: the current zero cutoff used by the reaction deletion computer.

---


### getObjectiveFunction

```
public MathExpr<java.lang.String> getObjectiveFunction()
```

:   Returns the current objective function.

    :   **Returns:**: the current objective function.

---


### getOptimizationSense

```
public OptimizationSense getOptimizationSense()
```

:   Returns the current optimization sense.

    :   **Returns:**: the current optimization sense.

---


### setObjectiveFunction

```
public void setObjectiveFunction(MathExpr<java.lang.String> objective)
```

:   Sets the objective function to the provided mathematical expression.

    :   **Parameters:**: `objective` - a mathematical expression of reaction names.

---


### setOptimizationSense

```
public void setOptimizationSense(OptimizationSense sense)
```

:   Sets the optimization sense to the provided value.

    :   **Parameters:**: `sense` - an optimization sense.

---


### setCatalystDelSource

```
public void setCatalystDelSource(java.lang.Iterable<java.util.Set<java.lang.String>> deletionSource)
```

:   Sets the catalyst deletion input source.

    :   **Parameters:**: `deletionSource` - a source of catalyst names sets whose effect upon deletion is to be computed.

---


### setZeroCutoff

```
public void setZeroCutoff(double zeroCutoff)
```

:   Sets the zero cutoff to the provided value. The zero cutoff is the value
    used to decide which computed values are close enough to zero to be
    considered equal to zero. This value is an upper bound and should itself
    be close to zero, like 1E-6 for example.

    :   **Parameters:**: `zeroCutoff` - the zero cutoff.

---


### getProgress

```
public ProgressMonitor getProgress()
```

:   Returns a progress monitor for this experiment.

    :   **Specified by:**: `getProgress` in interface `MonitoredProcess<FbaOptComputer.Results>`
    :   **Returns:**: a progress monitor for this experiment.

---


### run

```
public void run(ResultsManager<FbaOptComputer.Results> resultsManager)
```

:   Runs this process and adds the generated results to the provided results
    manager via its `ResultsManager.addData(Object)` method.

    A set of catalyst names is retrieved from the deletion source, their
    effect upon deletion is computed, and the results are added to the
    provided results manager. This is repeated until the end of the deletion
    source is reached.

    :   **Specified by:**: `run` in interface `KernelProcess<FbaOptComputer.Results>`
    :   **Parameters:**: `resultsManager` - the results manager to which the results will be added.

---


### getRxnNames

```
public static java.util.Set<java.lang.String> getRxnNames(java.util.Set<java.lang.String> catalysts,
                                                          IrrevRxnFluxome fluxome)
```

:   Returns the set of names of reactions whose activity depends on the
    provided set of catalysts.

    :   **Parameters:**: `catalysts` - a set of catalyst names.: `fluxome` - the fluxome containing the reaction-catalyst association. **Returns:**: the set of names of reactions whose activity depends on the provided set of catalysts.


---


|  |  |  |
| --- | --- | --- |
| **Overview** \| **Package** \| **Class** \| **Use** \| **Tree** \| **Deprecated** \| **Index** \| **Help** | |  |
| PREV CLASS   **NEXT CLASS** | **FRAMES**    **NO FRAMES**     **All Classes** |  |
| SUMMARY: NESTED \| FIELD \| CONSTR \| METHOD | DETAIL: FIELD \| CONSTR \| METHOD |  |


---
